# The Benefits of Friendships in Academic Settings: A Systematic Review and Meta-Analysis

**DOI:** 10.7759/cureus.50946

**Published:** 2023-12-22

**Authors:** Turki A Alotaibi, Khalid M Alkhalifah, Norah I Alhumaidan, Wijdan A Almutiri, Saad K Alsaleh, Faisal M AlRashdan, Hadeel R Almutairi, Ahmed Y Sabi, Abdullah N Almawash, Mayyasah Y Alfaifi, Majed Al-Mourgi

**Affiliations:** 1 College of Medicine, Taif University, Taif, SAU; 2 Unaizah College of Medicine and Medical Sciences, Qassim University, Unaizah, SAU; 3 College of Medicine, Princess Nourah Bint Abdulrahman University, Riyadh, SAU; 4 College of Medicine, Arabian Gulf University, Manama, BHR; 5 College of Medicine and Surgery, King Abdulaziz University, Jeddah, SAU; 6 College of Medicine, Jazan University, Baish, SAU; 7 College of Medicine, King Saud University, Riyadh, SAU; 8 Medicine and Surgery, King Abdulla Bin Abdulaziz University Hospital, Riyadh, SAU; 9 Department of Surgery, Medical College, Taif University, Taif, SAU

**Keywords:** systematic review and meta-analysis, gpa, academic performance, academic settings, friendships

## Abstract

Friendships can positively impact students' academic performance and grade point average (GPA) by providing emotional support and reducing stress, thereby leading to improved focus and better concentration on studies. Peer connections and friendships often result in collaborative learning and the exchange of academic ideas, improving comprehension and retention of course materials, ultimately leading to higher GPAs. In contrast, negative friendships or excessive social distractions can adversely affect GPA, which highlights the importance of striking a fine balance between social connections and academic responsibilities. This systematic review and meta-analysis adhered to the Preferred Reporting Items for Systematic Review and Meta-Analysis (PRISMA) guidelines. It involved a thorough electronic search on PubMed, Ebsco, and Web of Science databases with no time restrictions.

We considered studies from various parts of the world, which examined friendships and relations within the academic setting. This review delved into the substantial impact of friendships in academic settings. Friendships foster a supportive environment for collaboration and knowledge-sharing, ultimately enhancing motivation, reducing stress, and improving GPA, thereby contributing to a successful academic experience. While disparities were noted across studies due to geographical variations, study designs, and outcome measures, the majority of them revealed a positive correlation between friendship and academic performance. Some studies highlighted gender-related differences, with male friendships often proving beneficial for academic performance, though this is not a universal rule, as the quality of friendships mostly depends on compatibility rather than gender. To sum up, the extensive review of research underscores the pivotal role of friendships in academic settings, which act as crucial support systems for collaboration, knowledge-sharing, and motivation among students, leading to enhanced academic performance. Despite regional and methodological variations, a consistent positive correlation between friendship and academic success was observed across diverse studies.

## Introduction and background

Academic success is assessed by gauging academic performance in the form of grade point average (GPA), test scores, and overall academic achievement, as well as the measurement of academic motivation and the level of persistence among students in school or college. Forming friendships with their peers is an important aspect of adolescents' and young adults' lives, and significant research has been conducted on how friends impact academic performance and motivation. Specifically, academic achievement and motivation have been found to positively correlate with belonging to a peer group [1].

Currently, young people's need for a sense of community is particularly high [2], leading individuals to spend more time with their friends, feel more comfortable around friends than they do with family, and worry about how their friends will perceive them and how the local social milieu will view them [2]. Researchers' concerns about how social networking sites affect different aspects of life, including education, are not surprising. Academic achievement has been linked favorably to social connections or peer interactions in the past [3-5]. Indeed, empirical research has demonstrated the value of friendships for academic success. Some other researchers have narrowed their focus to peer networks, where teen interactions occur more frequently and in a variety of situations, since evidence for peer effects from classroom-based studies was inconsistent. Due to its growing significance as children progress from the early school years into adolescence, the subject of friendship has received considerable attention.

Establishing close friendships is considered to mark a significant developmental milestone during adolescence because it enables people to gain a deeper understanding of others and themselves through private, open, and vulnerable interactions [6]. According to studies conducted in middle and high schools [7], most students grow a "wider circle" of intricate, varied, intermediate social links with their peers during adolescence. This occurs both within and outside of the classroom. Children and teenagers interact with their peers regularly outside of the school setting through activities such as organized summer activities, extracurricular athletics, as well as unorganized sports, games, and hanging out with friends and friends of friends [8]. Middle and high school students interact with various peer groups within the context of the school through a variety of classroom settings, before- and after-school activities, and various structured, school-organized extracurricular activities, such as team and individual athletic activities, and academic clubs [8].

Researchers claim that the correlation between relationships and performance may hinge on students choosing their peers based on performance compatibility or resemblance. Hence, successful students may favor relationships with other successful students [9]. However, Bond et al. reported a mismatch in terms of friendship-related effects. While friendship among average and high-performing students showed no correlation to students' academic achievement, close friends who performed poorly in school showed an association. On the other hand, there is substantial evidence that peers have a major impact on learning, behavior, and enjoyment [10].

Exponential random graph models (ERGM) and permutation tests are two unique statistical methods that have been created for inferential analysis of empirical network data [11]. Permutation-based tests are effective and reliable tools for investigating how the network affects individuals or how the outcomes are affected by relationships or positions [12]. These findings naturally prompt the next question: how does a student's social network environment affect his or her academic success over time? Most of these assertions are supported by studies where the predictor variables for friends and individual outcome variables belong to the same domain. In other words, an individual's academic performance may be influenced by one of their friends' academic achievements, or their social behavior may have an impact on a particular aspect of their own social behavior. Researchers believe that to explain students' academic progress, one must consider their social relationships at school. From a social capital perspective, adolescent social ties at school provide them with the tools and encouragement they need to succeed in school [13].

Friendship in an academic setting fosters a supportive environment, encouraging collaboration and knowledge exchange, ultimately enhancing learning experiences [14,15]. On the other hand, friendships can sometimes lead to distractions in class. Students might engage in conversations or activities with friends during classes, causing disruptions in their focus on studies [15]. A systematic review and meta-analysis titled "Friends with performance benefits" asserts that friendship creates an excellent environment to perform optimally and improve academic outcomes [16]. In today's educational environment, marked by the prevalence of online learning platforms and heightened concerns about student well-being, understanding how friendships contribute to academic success is of paramount importance. In light of this, this review aims to consolidate and analyze existing research while considering methodological variations across studies. It provides a comprehensive understanding of the intricate relationship between friendships and academic achievement, offering valuable insights for educators, policymakers, and researchers.

## Review

Methods

Literature Search Strategy

This systematic review and meta-analysis was conducted by adhering to the Preferred Reporting Items for Systematic Review and Meta-Analysis (PRISMA) guidelines. A broad electronic search was conducted through PubMed, Ebsco, and Web of Science databases with no time restrictions. The search strategy involved using the terms "GPA (AND Friendship OR Friendships)" to identify all studies related to the benefits of friendships in academic settings globally.

Inclusion and Exclusion Criteria

We included studies conducted in academic settings, including elementary schools, middle schools, high schools, colleges, or universities; studies that examine the benefits of friendships on academic outcomes or academic motivation; studies published in the English language; and all study designs that met our criteria except systematic reviews. Exclusion criteria included studies conducted in non-academic settings, such as community or non-educational environments; studies that do not specifically focus on the benefits of friendships in terms of academic outcomes or related variables; studies published in languages other than English; and studies that were systematic reviews.

Selection of Articles and Data Extraction

All the articles yielded from the primary search were imported to Mendeley reference manager software for the removal of duplicates. After deduplication, the studies were imported to the Rayyan platform, and four authors (N.A., W.A., F.A., S.A.) independently screened the studies based on titles and abstracts and then reviewed the full texts of all the studies. Disagreements at any step of the screening process were resolved through debate and consensus among all authors. Extracted data from the studies included the name of the main author, year of publication, reference, country of origin, study design, sample size, age and sex of the participants, inclusion and exclusion criteria, nationality, friendship variables, academic outcomes, and conclusion.

Quality Assessment

The assessment of study quality for eligible papers was conducted using the Newcastle-Ottawa Scale. The assessment focused on three key criteria: the selection of study groups, the comparability of these groups, and the ascertainment of the outcome. To ensure the reliability and validity of this quality assessment, two independent investigators were involved in the process. In cases where discrepancies or disagreements arose during the assessment, a systematic approach was employed for resolution. This involved either reaching a consensus between the two investigators or collaborating with a third investigator to arrive at a final quality rating.

Statistical Analysis

We carried out an in-depth analysis of the data, calculating both individual odds ratios (OR) and pooled OR from the different studies. We visually presented the results using forest plots with 95% confidence intervals (CI). To determine the level of statistical heterogeneity across the included studies, we adopted Cochran's Q test and I^2^ statistics. These statistical methods were imperative in evaluating the degree of variability or inconsistency in the data. Furthermore, we examined the presence of publication bias in the outcomes by using funnel plots. Any noticeable asymmetry in these plots was considered an indicator of potential publication bias.

Results

**Figure 1:**
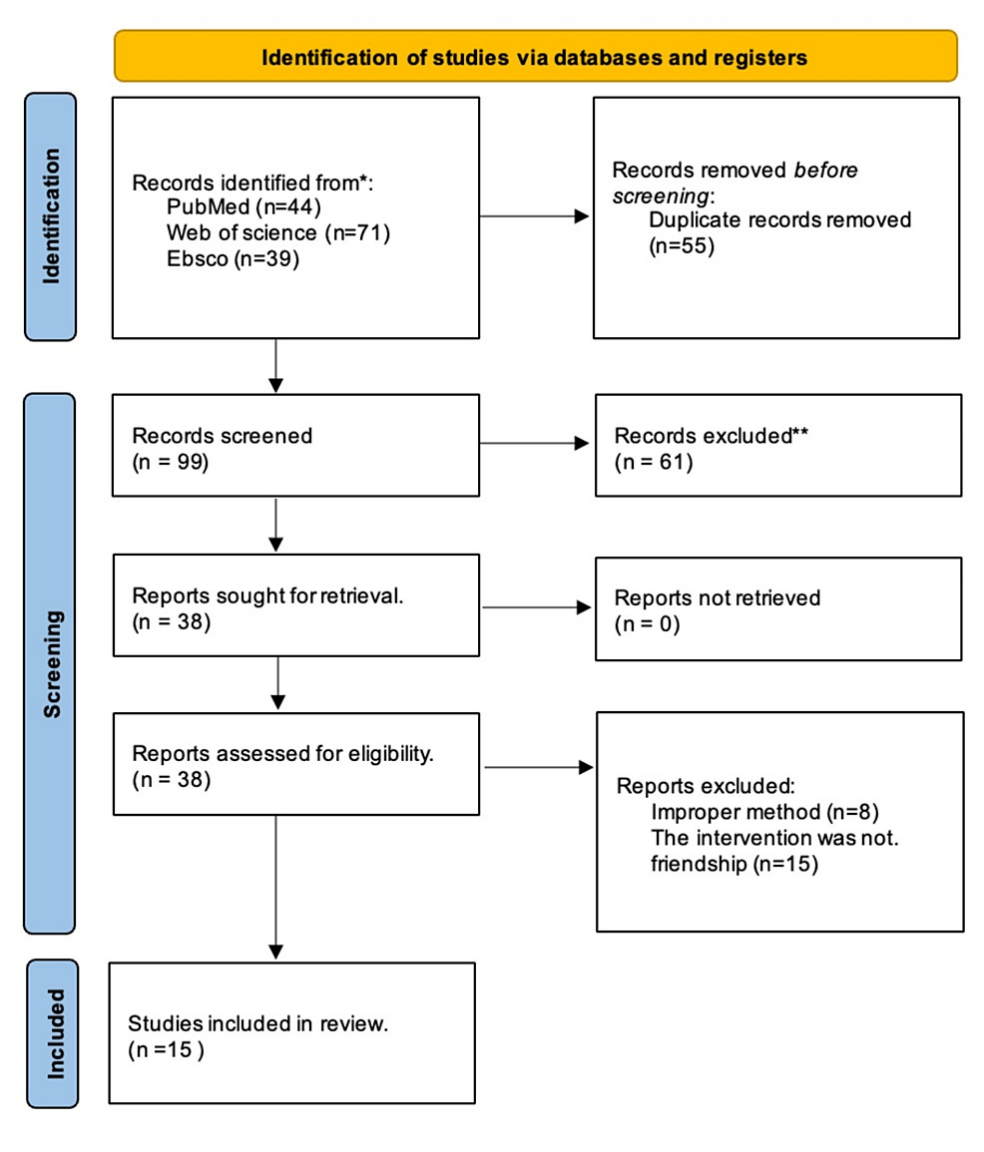
PRISMA flowchart depicting the selection of studies PRISMA: Preferred Reporting Items for Systematic Review and Meta-Analysis

Our initial search across the mentioned databases, as illustrated in Figure 1, yielded a total of 154 studies related to the topic of "benefits of friendships in academic settings." To ensure data accuracy, 55 duplicate records were meticulously removed before the screening process. Subsequently, the remaining 99 articles underwent a comprehensive screening procedure, which led to the exclusion of a further 61 records. The rigorous application of our inclusion and exclusion criteria led us to retrieve and assess 38 articles for eligibility. Among these, 23 studies failed to meet the specified selection criteria and were consequently excluded. A total of 15 articles that met our criteria and were deemed suitable for this systematic review were ultimately included in our review.

Study Characteristics

A total of 15 studies were selected from different regions and countries. There was significant diversity in terms of geographical area, age, sample size, and inclusion of subjects. The variation is illustrated in Table 1 below, based on country, study design, exposure, and academic outcomes. Most studies adopted a cross-sectional study design while others used either a longitudinal research design or a retrospective one. The sample sizes varied significantly, ranging from 119 to 10,369. Most of the studies used the GPA as a benchmark to determine whether friendship impacted academic performance with a few delving into specific subjects being studied in school. 

**Table 1:** Characteristics of the included studies GPA: grade point average; ECA: extracurricular activity

Author	Country	Study design	Sample size	Exposure	Academic outcomes	Conclusion (main findings)
Dodeen and Hamzeh, 2019 [17]	UAE	Cross-sectional	1,431	Social relationships	Academic performance (GPA)	Lonely college students scored below average, around 15% of them experienced loneliness
Hommes et al., 2012 [18]	Netherlands	Cross-sectional	301	Social interaction	Academic performance (GPA)	All three social networks increased student learning significantly, even after controlling for academic motivation, social integration, age, gender, and prior performance
Carbonaro and Workman, 2016 [19]	USA	Retrospective cohort	10,369	Students' friendship	GPA, college expectation, high school dropout, high school completion	Students' friends' GPA can be used to predict GPA (but not friends of friends). Students' friends (and friends of friends) dropping out increases the likelihood that the students will drop out
Kahalon et al., 2022 [20]	Israel	Longitudinal	2,304	Intergroup contact	Academic performance (GPA)	Arab students who participated in the course had a higher GPA than those who did not
Saqr et al., 2018 [21]	Saudi Arabia	Cross-sectional	119	Peer interactions	GPA, the difference between grades	There was a positive association between male networks and academic performance; however, no statistical association was found between female networks and performance
Hill, 2015 [22]	USA	Retrospective cohort	8,435	Opposite gender friendship	GPA and the expected educational and life outcomes	This study concluded that having a greater number of friends of the opposite gender is associated with a lower high school GPA for students
Furrer and Marchand, 2020 [23]	USA	Cross-sectional	443	Peer relationships	GPA, academic engagement	Students possessing abundant resources and few liabilities demonstrated elevated levels of self-reported and teacher-reported academic engagement, as well as higher GPAs in both the fall and spring terms. Conversely, the three hybrid profiles were linked to inferior academic performance
Corno et al., 2022 [24]	South Africa	Longitudinal	422	Interracial interaction	Total number of exams passed and failed and their GPA. Also, the academic evaluation by the faculty examination committees	Living with a racially diverse roommate fosters positive racial attitudes and academic success, benefiting both white and black students
Fujiyama et al., 2021 [25]	USA	Retrospective cohort	4,187	Peer relationships	Self-reported grades in four subjects (English, Science, Math, and Social Studies) on a four-point scale (A = 4, B = 3, C = 2, D or lower = 1)	There was a positive correlation between the average friend GPA and student GPA. Additionally, as expected, ECA member GPAs exhibited an asymmetric relationship with student GPAs
Goguen et al., 2020 [26]	USA	Cross-sectional	271	Peer relationships	Performance: GPA (fall and spring semesters, cumulative GPA), motivation: student persistence in college	Trust, shared interests, and the level of conflict with a new college friend were linked to both better GPAs and the likelihood of continuing into the second year of college
Cook et al., 2007 [27]	USA	Longitudinal	901	School friendships	Academic performance (GPA, math test scores, algebra placement)	Most friendship effects are specific to domains, with an individual's friends' GPA standing out as the most influential single attribute within the realm of friendships
Witkow, and Fuligni, 2020 [28]	USA	Longitudinal	629	In-school friendship	Academic performance (GPA)	Adolescents who had a greater number of friends within their school environment, as opposed to those outside of it, tended to have higher GPAs
Blansky et al., 2013 [29]	USA	Cross-sectional	158	Social network influences	Academic performance (GPA)	Students whose friends had higher (or lower) GPAs than their own showed a propensity for increasing (or decreasing) their academic ranking over time, revealing the presence of academic success contagion in their social network
Delgado et al., 2016 [30]	USA	Longitudinal	6,782	Adolescent friendship	Academic performance (GPA)	Friendships can play a crucial role in fostering a sense of school belonging, especially among highly marginalized groups within the United States, such as individuals of Mexican origin
Shin and Ryan, 2014 [31]	USA	Longitudinal	587	Adolescent friendship	Academic performance (GPA), academic motivation (persistence)	Friendships are influential on early adolescents’ motivation, engagement, and achievement in the classroom; being friends with a high achiever is likely to bolster a student’s grades, and being friends with a low achiever is likely to dampen a student’s grades

Forest Plot

**Figure 2:**
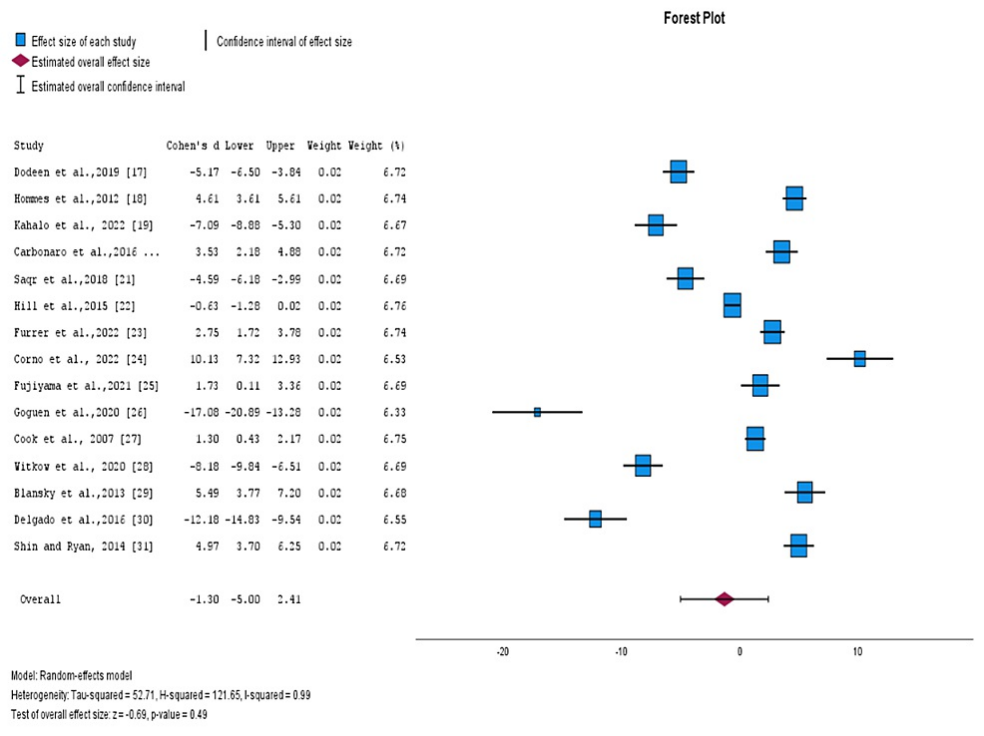
Forest plot

Figure 2 shows a forest plot that displays the effect sizes and confidence intervals of the effect sizes from each study, along with an overall summary estimate. The heterogeneity was measured to determine whether variability or diversity existed among the results of the different studies analyzed. We found that there was no significant heterogeneity across all studies, as shown by an I^2^ value of 99%. Hence there were no differences among the studies that might influence the overall interpretation. Notably, all studies were evenly weighted.

Funnel Plot

**Figure 3:**
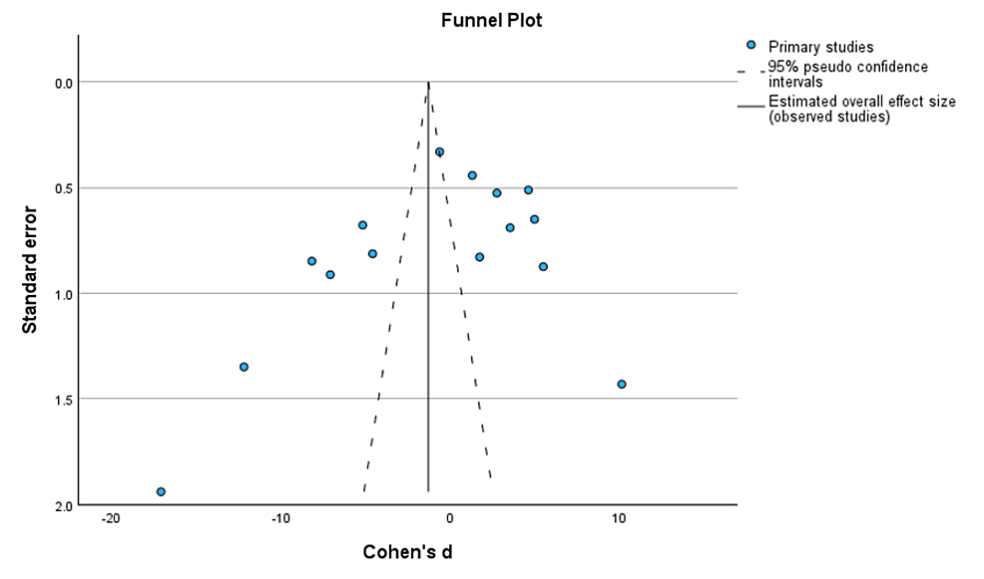
Funnel plot summarizing individual studies

Figure 3 shows a funnel plot used to assess the presence of publication bias while determining the presence of small-study effects. The plot below displays the association between the effect estimated from individual studies and the study precision. Upon examination of the funnel plot, asymmetry was evident, indicating potential publication bias.

Discussion

Friendships in academic settings can significantly impact student performance by fostering a supportive environment that encourages collaboration and knowledge-sharing. Building robust social bonds among classmates within educational institutions can bolster motivation, reduce stress, and result in an elevated GPA, ultimately creating a more conducive learning environment [17]. This systematic meta-analysis analyzed different aspects of friendships among different groups. Significant disparities were evident in the studies owing to differences in geographical area, study designs, and how academic outcomes were measured. However, most studies revealed a positive correlation between friendship and academic performance. Dodeen and Hassan conducted a study involving 1,429 tertiary students in UAE and found that there was no statistically significant relationship between loneliness and GPA scores [17]. On the contrary, a cross-sectional study by Hommes et al. among medical students revealed that social networks were positively associated with improved learning and better GPA [18].

A study conducted in the US by Carbonaro and Workman revealed exciting insights; their findings indicated that friends that students made in school were key predictors of academic expectations and impacted the risk of dropping out of school [19,20]. A similar study conducted in the same country discovered a positive correlation between the average friend GPA and student GPA, further revealing the impact of peer relationships. Various studies across the globe in our systematic review also concurred with this finding [20,21].

Only a few studies have been conducted about opposite-gender friendships in the academic setting. One study by Saqr et al. ascertained that male friendship in school was beneficial in terms of improved academic performance but not among females [21,22]. These findings, however, do not have a solid foundation as the dynamics vary greatly among individuals and are not determined solely by gender. The quality of any friendship depends on personal compatibility, shared interests, and mutual support rather than gender-based generalizations [23,24]. Another study that aimed to assess the effect of opposite-sex relationships in schools found that having friendships with the opposite gender significantly lowered the GPA among high school students, especially in mathematics and science [24].

Peer interactions between different racial groups foster diversity and inclusivity [25]. They enable individuals to learn from different perspectives, break down racial stereotypes, and allow students to learn and appreciate others. Corno et al. found that placing students from different races as room roommates was imperative in improving interracial interactions [26]. The study also revealed that black students who lived with white students improved their GPAs, and had lower dropout rates. A similar study from the US also revealed that friendships can play a crucial role in fostering a sense of belonging in schools, especially among highly marginalized groups within the US, such as individuals of Mexican origin [27].

The study by Witkow and Fuligni found that teenagers who had a larger circle of friends in school, as opposed to outside school, tended to achieve higher GPAs. Additionally, adolescents with higher GPAs tended to have more friends within the school environment. These connections were influenced by academic experiences, particularly those that were shared among friends [28]. This highlights the social dimension of education and the importance of peer dynamics in shaping motivation, engagement, and achievement among students. This is consistent with a cross-sectional study by Blansky et al., which showed a compelling connection between students' academic progress and the GPA averages of their friends [29]. When students surrounded themselves with friends who had a higher GPA average than their own, they exhibited a greater likelihood of improving their academic ranking over time. This phenomenon points to the existence of a social contagion effect within their social network, where academic success spreads among peers [29-30]. This positive correlation between friends' GPA and academic progress underscores the powerful influence of peer relationships on students' educational achievements, emphasizing the role of social networks in shaping academic outcomes [31].

We believe this study makes a significant contribution to the existing body of knowledge on the impact of friendships in academic settings. It combines various results from different studies across the world and study designs, highlighting the positive correlation between friendships and academic performance. Moreover, this study deepens our understanding of how friendships impact academic performance by considering various factors like gender dynamics, diversity in peer interactions, and the influence of peer groups on individual achievement.

## Conclusions

The extensive body of research examined in this review consistently reveals the pivotal role of friendships in academic settings. Friendships are fundamental support systems that encourage collaboration, knowledge-sharing, and motivation among students, ultimately contributing to improved academic performance. While some regional and methodological disparities exist in the literature, a predominant positive correlation between friendship and academic success is illustrated. The findings from various studies, spanning diverse geographical areas and academic contexts, consistently emphasize the profound impact of peer relationships on educational outcomes. Moreover, the influence of friendships transcends gender stereotypes, emphasizing that the quality of these relationships hinges more on personal compatibility, shared interests, and mutual support than on gender-based generalizations. The integration of students from different racial backgrounds further illustrates the potential of peer interactions to foster inclusivity and break down stereotypes. To conclude, the wealth of evidence supports the notion that friendships are not just a social aspect of education but a critical factor in students' overall academic experience.

## References

[1] Năstasă LE, Cocoradă E, Vorovencii I, Curtu AL (2022). Academic success, emotional intelligence, well-being and resilience of first-year forestry students. Forests.

[2] Orben A, Tomova L, Blakemore SJ (2020). The effects of social deprivation on adolescent development and mental health. Lancet Child Adolesc Health.

[3] Kolhar M, Kazi RN, Alameen A (2021). Effect of social media use on learning, social interactions, and sleep duration among university students. Saudi J Biol Sci.

[4] Tomé G, Matos M, Simões C, Diniz JA, Camacho I (2012). How can peer group influence the behavior of adolescents: explanatory model. Glob J Health Sci.

[5] Baldwin TT, Bedell MD, Johnson JL (1997). The social fabric of a team-based MBA program: network effects on student satisfaction and performance. Acad Manag J.

[6] Berndt TJ, Keefe K (1995). Friend’s influence on adolescent’s adjustment to school. Child Dev.

[7] Kim J, Fletcher JM (2018). The influence of classmates on adolescent criminal activities in the United States. Deviant Behav.

[8] De Meester A, Aelterman N, Cardon G, De Bourdeaudhuij I, Haerens L (2014). Extracurricular school-based sports as a motivating vehicle for sports participation in youth: a cross-sectional study. Int J Behav Nutr Phys Act.

[9] Brechwald WA, Prinstein MJ (2011). Beyond homophily: a decade of advances in understanding peer influence processes. J Res Adolesc.

[10] Bond RM, Chykina V, Jones JJ (2017). Social network effects on academic achievement. Soc Sci J.

[11] Robins G, Pattison P, Kalish Y, Lusher D (2007). An introduction to exponential random graph (p*) models for social networks. Soc Netw.

[12] Farine DR, Carter GG (2022). Permutation tests for hypothesis testing with animal social network data: problems and potential solutions. Methods Ecol Evol.

[13] Crosnoe R, Cavanagh S, Elder GH (2003). Adolescent friendships as academic resources: the intersection of friendship, race, and school disadvantage. Sociol Perspect.

[14] Wentzel KR (1998). Social relationships and motivation in middle school: the role of parents, teachers, and peers. J Educ Psychol.

[15] Kahu ER (2013). Framing student engagement in higher education. Stud High Educ.

[16] Chung S, Lount RB Jr, Park HM, Park ES (2018). Friends with performance benefits: a meta-analysis on the relationship between friendship and group performance. Pers Soc Psychol Bull.

[17] Dodeen H, Hassan A (2021). Assessing loneliness in UAE populations: the relationship with age, gender, marital status, and academic performance. Appl Res Qual Life.

[18] Hommes J, Rienties B, de Grave W, Bos G, Schuwirth L, Scherpbier A (2012). Visualising the invisible: a network approach to reveal the informal social side of student learning. Adv Health Sci Educ Theory Pract.

[19] Carbonaro W, Workman J (2016). Intermediate peer contexts and educational outcomes: do the friends of students' friends matter?. Soc Sci Res.

[20] Kahalon R, Shnabel N, Sharvit K, Halabi S, Wright SC (2023). High-quality contact with fellow majority group students is associated with better academic performance of minority group students. Pers Soc Psychol Bull.

[21] Saqr M, Nouri J, Fors U (2018). What shapes the communities of learners in a medical school. Edulearn Proc.

[22] Hill AJ (2015). The girl next door: the effect of opposite gender friends on high school achievement. Am Econ J Appl Econ.

[23] Furrer CJ, Marchand GC (2022). The adolescent peer system and academic engagement. Educ Psychol.

[24] Corno L, La Ferrara E, Burns J (2022). Interaction, stereotypes, and performance: evidence from South Africa. Am Econ Rev.

[25] Fujiyama H, Kamo Y, Schafer M (2021). Peer effects of friend and extracurricular activity networks on students' academic performance. Soc Sci Res.

[26] Goguen LM, Hiester MA, Nordstrom AH (2010). Associations among peer relationships, academic achievement, and persistence in college. J Coll Stud Ret.

[27] Cook TD, Deng Y, Morgano E (2007). Friendship influences during early adolescence: the special role of friends' grade point average. J Res Adolesc.

[28] Witkow MR, Fuligni AJ (2010). In‐school versus out‐of‐school friendships and academic achievement among an ethnically diverse sample of adolescents. J Res Adolesc.

[29] Blansky D, Kavanaugh C, Boothroyd C, Benson B, Gallagher J, Endress J, Sayama H (2013). Spread of academic success in a high school social network. PLoS One.

[30] Delgado MY, Ettekal AV, Simpkins SD, Schaefer DR (2016). How do my friends matter? Examining Latino adolescents’ friendships, school belonging, and academic achievement. J Youth Adolesc.

[31] Shin H, Ryan AM (2014). Early adolescent friendships and academic adjustment: examining selection and influence processes with longitudinal social network analysis. Dev Psychol.

